# Editorial: Fertility preservation

**DOI:** 10.3389/fgwh.2024.1452588

**Published:** 2024-08-12

**Authors:** Berna Dilbaz

**Affiliations:** Etlik Zubeyde Hanim Women's Health Training and Research Hospital, University of Health Sciences, Istanbul, Türkiye

**Keywords:** fertility preservation, premature ovarian failure, chemotherapy, ovarian teratoma, sexual education, sickle cell anemia

**Editorial on the Research Topic**
Fertility preservation

It gives me great pleasure to present this special issue entitled “*Fertility Preservation*” in “Frontiers in Global Women's Health” in the publication year 2023–2024. This issue consists of four articles, two from China and the remaining two from Brazil and the USA, respectively. Here are some summaries of the articles published in this issue of the journal. We hope you will enjoy reading these interesting articles.

One of the articles from China is a mini review by Li et al. on the research progress on prevention of premature ovarian failure (POF) caused by cisplatin treatment. POF is the depletion of ovarian follicles in women under the age of 40, leading to premature menopause. Patients usually present with infertility and amenorrhea as a result of hypergonadotropic hypogonadism with follicle-stimulating hormone levels above 40 IU/L and estradiol levels below 20 pg/ml. This condition leads to infertility, osteopenia, or osteoporosis and increases the risk of autoimmune and heart disease (1). A reduced quality of life and psychological problems such as stress and anxiety are other known consequences of this condition. Cisplatin has proven effects on DNA metabolism. This mini review describes premature ovarian failure and the mechanism of action of cisplatin, which is used as a chemotherapeutic agent. The vivid illustrations and brief summary of the etiology of premature ovarian failure are very clear and can be used in teaching. The authors summarize possible strategies that can be used to prevent cisplatin-induced premature ovarian failure.

An original study from Brazil by Bellotti et al. examines the surgical, oncologic, and obstetric outcomes of radical trachelectomy for early-stage cervical cancer (stage 1A2, stage 1B). Radical trachelectomy is a fertility-preserving surgical procedure that is usually offered to women of reproductive age for early-stage cervical cancer. During the removal of the cervix, the uterus is protected to preserve future fertility. This retrospective cohort study is based on data collected by the Brazilian National Cancer Institute Gynecologic Oncology Service. A total of 32 patients were recruited and the intra- and postoperative complications, obstetric outcomes, and pregnancy and mortality rates were analyzed in this article. The recurrence rate after radical trachelectomy was 3.1% and five out of 28 patients became pregnant during the follow-up period.

Sickle cell disease is a hereditary disease that affects the erythrocytes and can lead to multiple organ failure if left untreated. The disease itself has a deteriorating effect on the ovarian reserve, while the drugs used to treat it can also have a teratogenic effect, which is why contraception is recommended during treatment. In this cross-sectional study, Carrithers et al. investigated knowledge of infertility risk factors and perceptions of fertility treatment in adults with sickle cell disease, as fear of future infertility leads to rejection of sickle cell disease treatment.

The case report from China by Hu et al. on ovarian teratoma-associated anti-N-methyl-daspartate receptor encephalitis (anti-NMDAR) summarizes the five case series previously reported in the literature in addition to presenting this interesting case. The pathogenesis of teratoma-associated NMDA receptor encephalitis is still unknown. Surgical excision of the lesion, hormonal treatment, intravenous immunoglobulin, steroid therapy, and plasma exchange are the proposed treatment modalities, but an optimal treatment modality for ovarian teratoma-associated anti-N-methyl-daspartate receptor encephalitis is still lacking.

This special issue was met with great interest by our readers and was read and downloaded 4,941 times. We would like to thank the authors for their valuable scientific contribution. Developments in the diagnosis and treatment of serious diseases previously considered incurable or leading to infertility have opened the door to new approaches to fertility preservation. Fertility preservation has many aspects, ranging from oncofertility to legal and ethical factors. Advances in embryology, genetics, and assisted reproductive technologies are paving the way for cryopreservation of ovarian tissue, sperm, eggs, and embryos for fertility preservation. In this special issue, only some of these factors are discussed, in the hope that further challenges and innovations in this field will emerge and be discussed in the near future.
